# Inositol 1,4,5-trisphosphate receptor type 1 autoantibody (ITPR1-IgG/anti-Sj)-associated autoimmune cerebellar ataxia, encephalitis and peripheral neuropathy: review of the literature

**DOI:** 10.1186/s12974-022-02545-4

**Published:** 2022-07-30

**Authors:** Sven Jarius, Stefan Bräuninger, Ha-Yeun Chung, Christian Geis, Jürgen Haas, Lars Komorowski, Brigitte Wildemann, Christian Roth

**Affiliations:** 1 Molecular Neuroimmunology Group, Department of Neurology, University of Heidelberg, Heidelberg, Germany; 2 Department of Neurology, DRK-Kliniken Nordhessen, Kassel, Germany; 3 Section Translational Neuroimmunology, Department of Neurology, Jena University Hospital, Jena, Germany; 4 Institute for Experimental Immunology, affiliated to EUROIMMUN Medizinische Labordiagnostika AG, Lübeck, Germany

**Keywords:** Anti-neuronal autoantibodies, Inositol 1,4,5-trisphosphate receptor type 1 antibodies (ITPR1-IgG), Anti-Sj, Autoimmune encephalitis, Paraneoplastic neurological syndromes, Dementia, Cognitive decline, Cerebellar ataxia, Purkinje cell antibodies, Medusa head ataxia, Limbic encephalitis, Polyneuropathy, Cancer, IP3R1, InsP3R1

## Abstract

**Background:**

In 2014, we first described novel autoantibodies to the inositol 1,4,5-trisphosphate receptor type 1 (ITPR1-IgG/anti-Sj) in patients with autoimmune cerebellar ataxia (ACA) in this journal. Here, we provide a review of the available literature on ITPR1-IgG/anti-Sj, covering clinical and paraclinical presentation, tumour association, serological findings, and immunopathogenesis.

**Methods:**

Review of the peer-reviewed and PubMed-listed English language literature on ITPR1-IgG/anti-Sj. In addition, we provide an illustrative report on a new patient with ITPR1-IgG-associated encephalitis with cognitive decline and psychosis.

**Results:**

So far, at least 31 patients with serum ITPR1-IgG/anti-Sj have been identified (clinical information available for 21). The most common manifestations were ACA, encephalopathy with seizures, myelopathy, and (radiculo)neuropathy, including autonomic neuropathy. In 45% of cases, an underlying tumour was present, making the condition a facultative paraneoplastic neurological disorder. The neurological syndrome preceded tumour diagnosis in all but one case. In most cases, immunotherapy had only moderate or no effect. The association of ITPR1-IgG/anti-Sj with manifestations other than ACA is corroborated by the case of a 48-year-old woman with high-titre ITPR1-IgG/anti-Sj antibodies and rapid cognitive decline, affecting memory, attention and executive function, and psychotic manifestations, including hallucinations, investigated here in detail. FDG-PET revealed right-temporal glucose hypermetabolism compatible with limbic encephalitis. Interestingly, ITPR1-IgG/anti-Sj mainly belonged to the IgG2 subclass in both serum and cerebrospinal fluid (CSF) in this and further patients, while it was predominantly IgG1 in other patients, including those with more severe outcome, and remained detectable over the entire course of disease. Immunotherapy with intravenous methylprednisolone, plasma exchange, and intravenous immunoglobulins, was repeatedly followed by partial or complete recovery. Long-term treatment with cyclophosphamide was paralleled by relative stabilization, although the patient noted clinical worsening at the end of each treatment cycle.

**Conclusions:**

The spectrum of neurological manifestations associated with ITPR1 autoimmunity is broader than initially thought. Immunotherapy may be effective in some cases. Studies evaluating the frequency of ITPR1-IgG/anti-Sj in patients with cognitive decline and/or psychosis of unknown aetiology are warranted. Tumour screening is essential in patients presenting with ITPR1-IgG/anti-Sj.

## Introduction

**Fig. 1:**
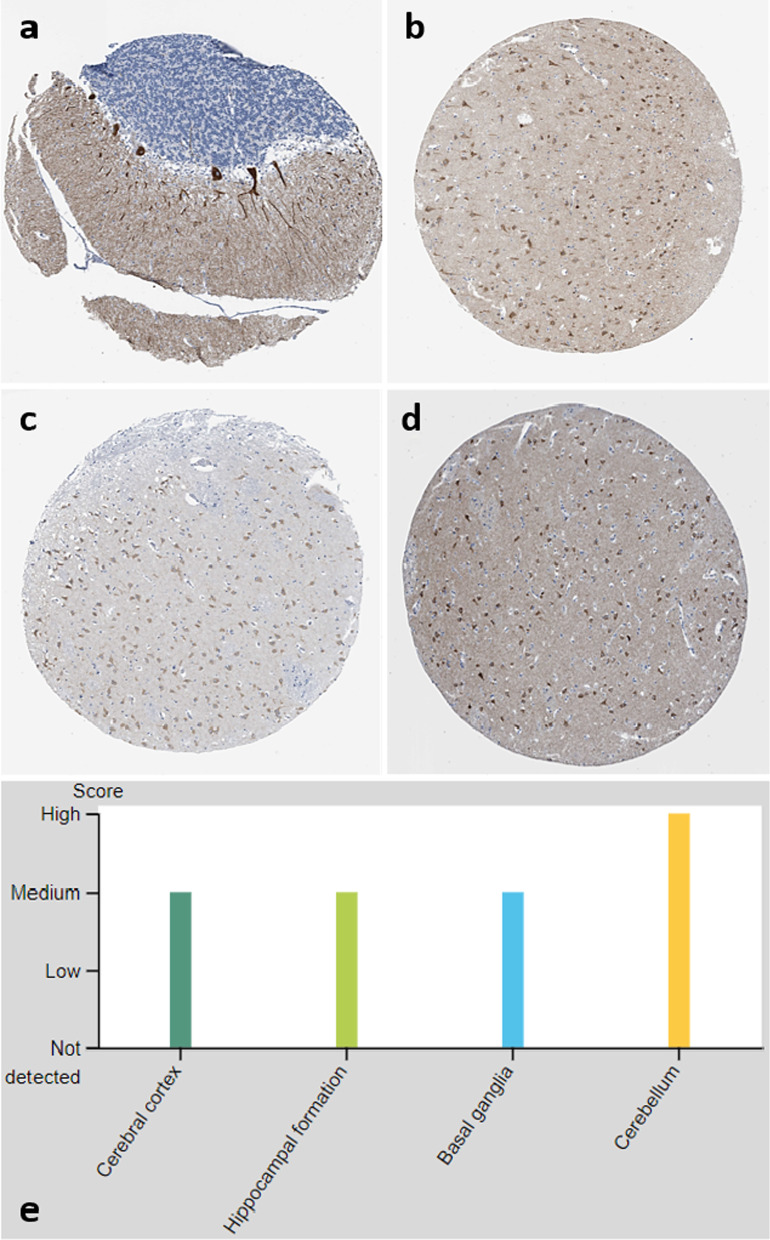
ITPR1 protein expression, as detected by immunohistochemistry, in the cerebellum (**a**), cerebral cortex (**b**), hippocampus (**c**), and lateral ventricle wall/basal ganglia (**d**), together with protein expression scores (**e**) (modified images from the Human Protein Atlas image database [40]; https://www.proteinatlas.org; licensed under the Creative Commons Attribution-ShareAlike 3.0 International License)

In 2014, we described in this journal novel immunoglobulin G (IgG) autoantibodies against the inositol 1,4,5-trisphosphate receptor type 1 (ITPR1; also termed IP3R1 or InsP3R1) in patients with autoimmune cerebellar ataxia [1]. The target antigen was identified by us in 2010 and the antibody initially termed anti-Sj after the index sample code. When tested by immunohistochemistry on rodent or primate cerebellum sections, IgG from serum and cerebrospinal fluid (CSF) samples of ITPR1-IgG/anti-Sj-positive patients characteristically bind to dendrites and cell bodies of Purkinje cells (PC). ITPR1-IgG/anti-Sj-associated ataxia thus forms part of a broader spectrum of anti-PC antibody-related ataxias referred to collectively as ‘medusa head ataxias’, based on the specific immunohistochemical staining pattern [2–4]. Many of these antibodies target proteins involved in intracellular calcium homoeostasis, such as anti-Homer-3 [5], anti-PKCγ [6], anti-mGluR1 [7, 8], anti-GluR-delta2 [9, 10], anti-CARPVIII [11, 12] and, possibly, anti-ARHGAP26/anti-Ca [13–15]. However, ITPR1 is also expressed in other central and peripheral neurons (Fig. 1) [16, 17], and, accordingly, we and others subsequently described ITPR1-IgG/anti-Sj antibodies also in patients with paraneoplastic and non-paraneoplastic (radiculo)neuropathy, autonomic neuropathy, myelopathy, possible brainstem encephalitis and encephalopathy with seizures [16, 18]. While ITPR1 is usually considered an intrathecal antigen, surface localization of ITPR1 has been reported to occur in some neurons and other cell types [19–25].

Here, we give a comprehensive review of all ITPR1-IgG/anti-Sj-seropositive cases published so far and discuss the immunopathophysiology of this rare syndrome. We include in the analysis a recent case in which ITPR1-IgG/anti-Sj was associated with progressive cognitive decline, affecting mainly short-term memory, executive dysfunction, attention deficits, a sleep disorder, and psychotic symptoms.

## Methods

### Literature review and case report

Cases were identified by a PubMed search including all English language articles on ITPR1-IgG/anti-Sj in patients with autoimmune encephalitis published between our first description of ITPR1-IgG in 2014 and 2022 using the following search term: (“inositol 1,4,5-trisphosphate receptor 1” OR ITPR1 OR IP3R1 OR InsP3R1) AND (antibody OR autoantibody OR antibodies OR autoantibodies OR “immunoglobulin G” OR IgG), and from the authors own files. Clinical and paraclinical data of a novel case of ITPR1-IgG/anti-Sj-associated autoimmune encephalitis were obtained retrospectively from the patient’s records. A descriptive statistical analysis of all data obtained was performed.

### Antibody testing

Immunohistochemistry (IHC) was performed as previously described [1]. Briefly, 4-μm cryosections of primate cerebellum (Euroimmun, Lübeck, Germany) were incubated for 1 h with diluted patient and control serum or cerebrospinal fluid (CSF) samples, respectively. Bound patient serum and CSF IgG was detected using goat secondary antibodies to human IgG, IgM and IgA, respectively, labelled with fluorescein isothiocyanate (FITC) (Euroimmun).

Specificity of the patient’s antibody response for ITPR1 was further confirmed using a fixed cell-based assay (CBA) as previously described [16]. Briefly, murine full-length ITPR1 was expressed in human embryonic kidney (HEK) 293 cells. HEK293 cells were then grown on sterile cover glasses for 48 h and subsequently fixed with acetone. Cover glasses were then cut into millimetre-sized fragments (biochips), which were used as assay substrate. Biochips with mock-transfected HEK293 cells were used as control substrate. Both substrates were incubated with the patients’ serum samples or control samples in parallel. Binding of patient serum and CSF IgG was visualized using a goat secondary antibody to human IgG labelled with FITC (Euroimmun).

For IgG subclass analyses, unconjugated sheep anti-human IgG antibodies specific for IgG subclasses 1 to 4 (The Binding Site, Schwetzingen, Germany) were substituted for the FITC-labelled goat anti-human IgG antibody and an Alexa Fluor^®^ 568-labelled donkey anti-sheep IgG secondary antibody (Invitrogen; absorbed against human IgG) was used to detect the IgG subclass-specific secondary antibodies, both in the IHC and in the CBA assay.

## Results

### Section A: Illustrative case report

We included in the analysis a new, so far unpublished case described in detail in the Appendix of this article.  A summary can be found in Table 1. ITPR1-IgG/anti-Sj was associated with progressive cognitive decline in this patient, affecting mainly short-term memory, executive dysfunction, attention deficits, a sleep disorder, and psychotic symptoms. Immunotherapy with high-dose intravenous methylprednisolone (IVMP) given during periods of acute deterioration, either alone or with plasma exchange (PLEX) and intravenous immunoglobulins (IVIG), repeatedly resulted in significant recovery. Long-term therapy with cyclophosphamide led to some stabilization. However, several relapse-like end-of-dose episodes occurred, characterized by either worsening of pre-existing symptoms or occurrence of new symptoms. Clinical and paraclinical data covering a period of more than 2 years, including serological, CSF, magnetic resonance imaging (MRI), and fluorodeoxyglucose positron emission tomography (PET) findings as well as neuropsychological test results, are provided in the Appendix.

**Table 1:** Clinical symptoms, laboratory and radiological findings and treatment in an illustrative case of ITPR1-IgG-positive encephalitis over a period of 2 years

	Aug–Sep 19	End Oct 19	Mid-Jan 20	Feb 20	Mar 20	End Mar 20	Beg-May 20	Jul 20	Sep 20	Oct 20	Nov 20	Jan 21	Apr 21	Jun 21	Mid-Jul 21	Aug 21
Clinical course	1st symptoms Aug 19 (incr. forgetfulness), rapid decline in short-term memory, confused actions, personal. changes Sep 19	Hosp. admiss.: dysdiad..; flat affect, irritab., persever., sev. cogn. impairm. (conc./attent.; short + long-term + working mem., process. speed, action plann., probl./error recogn.)→ “dysexec. dementia” → rapid deteror. with disor., impair. impulse contr., behav. abnorm	Sust. marked improvem. (normal. affect and exec. funct. with restored probl. recogn. + solution capac.) but mild resid. attent. deficit	Relapse with marked decline in cognitive function (see Appendix for details)	Marked improvem. of impulse contr., reduct. in psychomot. restlessn., but resid. impair. attent. + proc. speed	Newly emerging ataxia and mild tetraparesis	1st dose of CYCL→ further im- prov. of cogn. test results, but residually impaired attent., mem., and proc. speed	-	-	Relapse-like epis. ~ 1 mo after 3rd CYCL cycle, attent. + mem. defic., disorient., psychomot. restlessn. and formal thought disorder	Signific. neurocogn. improvem., compl. psychiatr. recovery Nov 20; followed by relat. stability Dec 20-Aug 21, but mild increase in symptom severity shortly before each CYCL cycle; episode of blurred vision in both eyes June 21 (lasting for 6–8 weeks, followed by complete recov.); re-admitted mid-Jul 21 with mild depress. disorder plus mild subj. decline in short-term mem. and exec. functions (prompt resolution after IVMP)					At last follow-up: resid. cogn.def.; gait instab. and limb ataxia mostly resolved but still uses rollator for resid. parapar
ITPR1-IgG CBA, serum	–	1:1000	1:1000	1:1000	–	–	1:100	–	–	–	1:320	–	–	–	–	1:1000
ITPR1-IgG CBA, CSF	–	1:1	–	1:4	–	–	–	–	–	–	–	–	–	–	–	–
ITPR1-IgG CBA, PLEX-E		–	–	1:320	–	–	–			–	–		–	–	–	–
ITPR1-IgG TBA, serum	-	1:320	1:1000	–	–	–	–	–	–	–	–	–	–	–	–	–
Other anti-neural Abs	–	Neg	Neg (serum + CSF)	Neg	–	–	–	–	–	–	–	–	–	–	–	–
CTD-ass. auto-Abs	–	Neg	–	Neg	–	–	–	–	–	–	–	–	–	–	–	–
CSF	–	Normal	Normal	Normal	–	–	–	–	–	–	–	–	–	–	QAlb ↑	–
CRP (mg/l; < 5)	–	7 → 7.7 → 9.3	13	Normal	–	–	–	–	–	13	–	–	–	–	–	–
BSR (mm/h; < 20)	–	22	–	Normal	–	–	–	–	–	–	–	–	–	–	–	–
CA125 (U/ml; < 35)	–	–	111	88	–	–	–	–	–	–	–	–	–	–	–	35
MMSE	–	27/30 → 23/30 → 29/30 at discharge end of Nov	29/30	24/30	28/30	–	30/30	–	–	–	–	–	–	–	–	–
Clock-drawing test	–	3/7	7/7	1/7	3/7	–	7/7	–	–	–	–	–	–	–	–	–
DemTect	–	9/18	16/18	8/16	14/18	–	15/18	–	–	–	–	–	–	–	–	–
EEG	–	2 × ri frontopar. delta activ	Ri par.-occ. theta activ	Ri par.-occ. theta activ	Normal	–	–	–	–	Intermittent ri-temp. slowing	Normal	–	–	–	–	–
cMRI	–	2 × normal (1 × with Gd)	Normal	Normal	-	–	–	–	–	Normal	–	–	–	–	Normal	–
sMRI	–	–	–	Normal	-	–	–	–	–	-	–	–	–	–	–	–
FDG-PET, head	–	–	–	Glucose metabol. ↑ ri med. temp lobe (amygd., parahipp.) + ri striatum, esp. putamen	-	–	–	–	–	-	–	–	–	–	–	–
FDG-PET, body	–	–	–	Normal	-	–	–	–	–	-	–	–	–	–	–	–
CT abd/thor/pelv	–	Bilat. small nodular abnormal. in the lung (< 6 mm), cystic structures at isthmus uteri	-	Normal (pelv.)	-	–	–	–	–	-	–	–	–	–	–	–
Acute treatment		IVMP (5 × 1 g)	IVMP (3 × 1 g)^a^, oral tapering	IVMP (5 × 1 g), 5 × PLEX, IVIG (4 × 35 g/day)	–	–	–	–	–	IVMP (5 × 1 g)^b^, oral tapering	–	–	–	–	IVMP (3 × 1 g)	–
Outcome from acute therapy	–	Almost complete resolution	Substantial improvement	Significant improvement	–	–	–	–	–	Significant improvement	–	–	–	–	Prompt resolution	–
Cyclophosphamide	–	–	–	–	–	–	CYCL (350 mg/ m^2^/d for 3 d)	CYCL	CYCL	-	CYCL	CYCL	CYCL	CYCL	-	CYCL

CBA, cell-based assay; cMRI, cranial MRI; BSR, blood sedimentation rate; CA125, cancer antigen 125; CSF, cerebrospinal fluid; CRP, C-reactive peptide; CT, computed tomography; CTD, connective tissue disorders; CYCL, cyclophosphamide; EEG, electroencephalography; FDG-PET, 2-deoxy-2-[18F]fluoro-d-glucose positron emission tomography; Gd, gadolinium; IVIG, intravenous immunoglobulins; IVMP, intravenous methylprednisolone; MMSE, mini mental status examination; MRI, magnetic resonance imaging; PLEX, plasma exchange; PLEX-E, PLEX eluate; QAlb, albumin CSF/serum ratio; sMRI, spinal MRI

^a^Plus risperidone, pipamperone, lorazepam

^b^Plus risperidone, lorazepam

### Section B: Review of the literature

Including the case described in Section A, 21 patients with serum antibodies to ITPR1 and central and peripheral neurological symptoms have been described in the literature so far (and at least 10 further patients have been identified in historic series of samples with anti-PC antibodies but not yet published due to the sparsity of available clinical data [4, 18]):

1. In 2014, we reported on ITPR1-IgG/anti-Sj in a woman with a 10-year history of progressive ataxia of the upper limbs, dysarthria, and gaze disturbances. MRI revealed pontocerebellar atrophy [1]. A follow-up report on this patient was published in 2017 by Berzero et al. [26]. Although the patient was positive for a BRCA1 (breast cancer 1, early onset) gene mutation and had a history of breast cancer on her mother’s side of the family, no tumour was initially found despite extensive diagnostic work-up, including regular screening with whole-body 18FDG-PET. Steroid treatment, ten cycles of PLEX, and three cycles of intravenous immunoglobulin, all first given several years after disease onset, had no significant beneficial effect with regard to disease progression. The disease came to a halt only when the patient was already severely disabled [1, 26]. Eleven years after onset, detection of suspicious lymph nodes upon mammography led to a diagnosis of bifocal ductal grade I breast cancer. Of note, immunopathology revealed substantial ITPR1 expression in the tumour and the metastatic lymph nodes [18]. Two further ITPR1-IgG/anti-Sj-seropositive patients investigated in the original 2014 report had “cerebral ataxia” and a “chronic cerebellar syndrome responsive to immunotherapy”, respectively, but no additional clinical information was available [1].
2. In 2016, we reported on serum ITPR1-IgG/anti-Sj in three male patients with (radiculo)polyneuropathy and, in one of them, neurogenic pain, consistent with the fact that ITPR1 has been shown to be expressed also in the anterior horn of the spinal cord, in the substantia gelatinosa, and in the motor, sensory (including the dorsal root ganglia) and autonomic peripheral nervous system. In two cases, the disease was associated with an ITPR1-expressing adenocarcinoma of the lung and multiple myeloma, respectively. Of note, ITPR1-IgG/anti-Sj serum titres declined after removal of the lung tumour, which was paralleled by clinical stabilization [16].
3. In 2017, three further ITPR1-IgG/anti-Sj-seropositive patients (two of them female) with cerebellar ataxia were described [27], one of whom also developed dysautonomia. One patient was successively treated with steroids, glatiramer acetate, and interferon-β-1a for suspected MS, none of which was convincingly effective in slowing disease progression (tumour status unknown), while no data on treatment outcome were available for the remaining patients (no evidence of paraneoplastic aetiology).
4. In 2018, a series of 11 (8 × female) patients with serum ITPR1-IgG/anti-Sj autoantibodies from the Mayo Clinic was published. Alfugham et al. [18] reported cerebellar ataxia in three cases, peripheral neuropathy in five (1 × autonomic), encephalitis with seizures in two, and myelopathy in two. Additional symptoms compatible with possible brainstem involvement included double vision and vertigo. In one patient, just as in the present case, visual blurring occurred. In five of the 11 patients (45%), ITPR1-IgG/anti-Sj was associated with cancer. In none of the seven patients receiving immunotherapy did the neurologic impairment improve significantly.
5. In 2022, a Chinese patient with ITPR1-IgG/anti-Sj-positive cerebellar ataxia associated with an adenocarcinoma of the lung and multiple brain metastases was described [28]. A broad screening for other paraneoplastic and non-paraneoplastic anti-neural antibodies was negative.

In addition to the cases summarized above, five further patients with ITPR1-IgG/anti-Sj detected only in the CSF have been reported (2 × seronegative [29, 30], 2 × no serum available [18], 1 × no information provided [18]). However, as all previously published ITPR1-IgG/anti-Sj-positive patients had serum antibodies to ITPR1, and the specificity of the recombinant assays used to detect ITPR1-IgG/anti-Sj has not been formally evaluated for CSF, the clinical significance of this finding is unknown. Moreover, co-existing CSF IgG antibodies to *N*-methyl d-aspartate receptors (NMDAR) (2×) and glial fibrillary astrocytic protein (GFAP) (3×) [16, 29, 30] were reportedly present in the CSF of some of these patients. CSF GFAP-IgG and CSF NMDAR-IgG can also cause autoimmune encephalomyelitis or encephalitis, respectively, rendering it impossible to attribute these patients’ clinical and paraclinical features to either of these antibodies with sufficient certainty. In one of these patients [30], ITPR1-IgG was negative in the CSF on three occasions, including at disease onset, but reportedly temporarily positive once, and negative in the serum in four matched serum samples, ITPR1-specific binding of the patient’s IgG to brain tissue was not demonstrated, and the associated tumour did not express ITPR1, which in summary casts doubts on the diagnosis. Accordingly, these cases were not included in this literature review. Of note, the new patient described in detail in Section A of the present article was negative for NMDAR-IgG and GFAP-IgG, as determined in three consecutive paired CSF and serum samples.

Very recently, a patient with suspected ITPR1-IgG seropositivity and confabulations, amnesia, and motor memory loss has been reported [31]. However, binding of IgG to ITPR1-IgG transfected cells was found only at an extremely low titre (1:10), and no corresponding, ITPR1-specific tissue staining was apparently observed. In consequence, doubts remain regarding the diagnosis also in this case. Accordingly, the case was not included in the statistical analysis.

#### Epidemiology

Ethnic origin was not reported for most patients (5 × Caucasian, not reported in the remainder, which included one report from a Chinese group); 9 patients were seen at European institutions; 11 at a North American institution; and one at a Chinese institution. The male:female ratio was 1:1.5. Interestingly, however, 6/7 patients with peripheral neuropathy were male. The median age at onset was 64 years (range 13–83), with no significant difference between patients with peripheral and patients with central nervous system involvement (median 60 years vs. 65 years).

Of particular interest, one study aimed at prospectively evaluating the frequency of ITPR1-IgG/anti-Sj among specimens submitted for paraneoplastic autoantibody evaluation, reporting a frequency of 15/100,000 specimens/year (although it is unclear whether this includes three of the four patients reportedly positive only in the CSF). This would be 15 times the frequency of anti-Tr/DNER (delta/notch-like epidermal growth factor-related receptor) (1/100,000/year) but lower than that of anti-Hu/ANNA-1 (100/100,000/year), anti-Ri/ANNA-2 (30/100,000/year) and anti-Yo/PCA-1 (80/100,000/year), based on a very large sample (N = 52,000) [18].

#### Cerebellar ataxia

Symptoms of cerebellar ataxia, either alone (N = 2) or in combination with other symptoms (N = 9), were present in 11/21 (52%) ITPR1-IgG/anti-Sj-seropositive patients, including limb ataxia with dysmetria, dysdiadochokinesis, intention tremor, gait ataxia, dysarthria, and gaze disturbances (Table 2). Co-existing symptoms in patients with cerebellar ataxia included double vision, visual blurring, subacute hearing loss, mild bilateral lower limb weakness, dysautonomia (2×), REM sleep disturbances, vertigo (2×), dysphasia, agraphia, and cognitive decline and other neuropsychiatric symptoms (Table 2).

**Table 2:** ITPR1-IgG-seropositive patients reported in the English language literature: sex, age, clinical phenotypes, MRI features, tumour association

No.	Ref	Sex	Age	Clinical phenotype	Associated tumour	MRI
*a. With tumour diagnosis at last follow-up*						
1	[1, 26]	f	28	ACA (upper limb ataxia, severe gait ataxia, dysarthria, gaze disturbances)	Breast cancer, metastatic lymphadenopathy	Pontocerebellar atrophy with hot cross bun sign, w/o any other supra- or infratent. abnormalities
2	[16]	m	60	PN (subacute motor axonal and demyelinating, EMG+), plus dysautonomia and impaired bladder/bowel function	Lung cancer (pT1b pN0 [0/18] L0 V0 Pn0 G2 R0)	Normal (cMRI + sMRI), later stroke
3	[16]	m	40	PN (sensory), plus depression	Multiple myeloma	N.d
4	[18]	m	72	PN (demyel. with sens. ataxia, EMG+)	Renal cell cancer, bone + liver metastases	N.d
5	[18]	f	60	PN (subacute, progressive axonal sensory > motor polyneuropathy, EMG+), plus myelopathy	Breast cancer, axillary lymph adenopathy	LETM (T2), diffuse enhancement of cervical nerve roots
6	[18]	f	83	Encephalitis (encephalopathy with EEG seizures, cogn. decl.), plus subacute pandysautonomia (TST, ART)	Lung cancer, mediastinal lymphadenopathy	Generalized brain atrophy, prominent in temporal lobes
7	[18]	f	65	ACA, plus double vision, subacute hearing loss	Breast cancer, malignant cells in the CSF	Normal
8	[18]	f	13	Myelitis (subacute spastic paraparesis)	High cancer/leukaemia risk due to Fanconi anaemia	Gd+ lesions of cerebellum, spinal cord, supratentorial brain; meningeal enhancement
9	[18]	f	65	Cervical dystonia, insomnia, anxiety, depression	Breast cancer, endometrial cancer	N.d
10	[28]	m	66	ACA (mild left cerebellar signs with dysmetria), plus mild bilateral lower limb weakness	Lung cancer (adenocarcinoma)	Brain metastasis
*b. No tumour identified at last follow-up*						
11	[1]	N.d	N.d	ACA	N.d	N.d
12	[1]	N.d	N.d	ACA	N.d	N.d
13	[16]	m	79	PN (slowly progressive sensory and motor axonal and demyelinating, EMG+)	No tumour known	Normal (sMRI)
14	[27]	m	41	ACA (gait ataxia, horizontal nystagmus, bilateral intention tremor)	No tumour known	Several demyelinating brain lesions (Gd−)
15	[27]	f	42	ACA (gait ataxia, horizontal nystagmus, dysarthria, dysmetria, UL dysdiadochokinesis), plus dysautonomia	No tumour known	Mild cerebellar atrophy
16	[27]	f	79	ACA (gait ataxia, ataxia of UL, dysarthria), plus dysautonomia (orthostatic hypotension) and REM sleep disturbances	No tumour known	Multiple ischaemic lesions
17	[18]	m	64	PN (subacute painful symmetric diffuse axonal neuropathy, EMG+)	No tumour known (PET–CT)	N.d
18	[18]	f	71	ACA, plus vertigo, dysphasia, agraphia	No tumour known, cervical dysplasia	Cerebellar atrophy
19	[18]	f	64	ACA, plus vertigo, visual blurring	No tumour known, severe weight loss	N.d
20	[18]	f	83	Encephalitis with acute status epilepticus	No tumour known (PET–CT)	Subcortical right occipito-parietal T2 signal (Gd+)
21	Present case	f	48	ACA, plus encephalitis (rapidly progress., severe cogn. decline, mainly affect memory, attent. and execut. functions, optic hallucin., depression)	No tumour known, but elevated CA125	Brain MRI normal, but FDG-PET showed glucose hypermetabolism in the right medial temporal lobe (amygdala, parahippocampus) and basal ganglia

ACA, autoimmune cerebellar ataxia; ART, autonomic response testing; CA125, cancer antigen 125; cMRI, cranial MRI; EMG, electromyography; Gd, gadolinium; LETM, longitudinally extensive transverse myelitis; MRI, magnetic resonance imaging; N.d., no data; PET–CT, combined positron emission tomography and computed tomography; PN, peripheral neuropathy; REM, rapid eye movements; sMRI, spinal MRI; TST, thermoregulatory sweat test

#### Cognitive decline and other neuropsychiatric symptoms

In the patient described in Section A, ITPR1-IgG/anti-Sj seropositivity was associated with rapidly progressive, severe cognitive decline, mainly affecting memory, attention and executive functions; optic hallucinations; and depression. A further patient originally presented with subacute pandysautonomia but developed dementia, encephalopathy and “electrographic seizures” 4 years later. MRI showed generalized brain atrophy, affecting particularly the temporal lobes [18]. The patient initially responded well to steroid treatment but the disease subsequently progressed. FDG-PET/CT suggested a lung tumour with mediastinal lymphadenopathy. ITPR1-IgG/anti-Sj was detected at a very high serum titre of 1:30,720 and a high CSF titre of 1:128. Of note, signs of possible limbic involvement (confusion, severe memory loss, suspected temporal lobe epilepsy) were also present in two ITPR1-IgG/anti-Sj-seropositive patients identified by us in 2016, one of whom also had cerebellar ataxia. As no detailed clinical data are available, the cases of these two patients have not yet been published (but were briefly mentioned in [16]). In another patient, who had breast and endometrial carcinomas, insomnia, anxiety and depression were reported, but no cognitive decline [18]. Severe depression of unknown cause developed also in an ITPR1-IgG/anti-Sj-seropositive patient reported in reference [16].

#### Sleep disorders

Sleep disorders have recently been described in association with a number of autoantibody-associated CNS disorders, including anti-IgLON5 [32, 33], anti-DPPX [34–36], anti-CASPR2 and anti-NMDAR encephalitis [37]. It should therefore not go unmentioned that the patient described in Section A reported hypersomnia at onset. When, later, psychomotor restlessness occurred, treatment with pipamperone was started (20 mg/day) in February 2020 and intensified (40 mg/day) in October 2020 because of increasing restlessness and hyposomnia. In January 2020 an attempt to withdraw pipamperone resulted in disruptions in sleep and inner unrest, and treatment was subsequently resumed at a reduced dose (20 mg/day). Sleep disorders were also present in at least two other previously described ITPR1-IgG/anti-Sj-seropositive patients, one with insomnia [18] and one with REM sleep disturbances [27].

#### Poly(radiculo)neuropathy

Electroneurography (ENG) indicated axonal and demyelinating polyneuropathy (PNP) in 2, axonal PNP in 1, and demyelinating PNP in 1 patient (ENG results not reported in 2 further patients). Both motor (at least 3 patients) and sensory (at least 6 patients) nerves were affected. The onset of PNP-associated symptoms was subacute in 4 and slowly progressive in 1 (no data in 2). Electromyography (EMG) corroborated the presence of PNP in all six cases with available data. In at least one case, clinical and electrophysiological findings suggested GBS [16]. In that patient, first severely delayed and missing F waves, which indicate damage to the proximal motor nerve axons or anterior horn α-motor neuron cell bodies, and A waves were noted, and, later, delayed motor nerve conduction velocities and decreased sensory nerve action potentials [16]. Interestingly, in this case also delayed motor neuron conduction velocity and distal motor latency of the facial nerve and dysautonomia was observed.

In another patient, also delayed and lost F waves were noted as well as A waves, decreased and lost compound muscle action potentials, missing sensory nerve action potentials and reduced sensory nerve conduction velocities in the upper and lower extremities and no conduction blocks. EMG suggested denervation [16].

#### Pain

Of note, most severe pain was present since onset in one of the patients with axonal and demyelinating peripheral neuropathy (ascending from the lower legs to the lumbar region) at an intensity that required treatment with tilidine, metamizole and gabapentin [16]. Pain was also a leading symptom a in second patient with EMG-confirmed symmetric diffuse axonal neuropathy [18]. We have previously pointed to the fact that ITPR1 is expressed in the sensory dorsal root ganglion, the trigeminal ganglia, and the substantia gelatinosa, in which C fibre axons synapse with neurons of the pain-transmitting lateral spinothalamic tract and damage to which can cause pain and hyperalgesia. Moreover, ITPR1 dysregulation was found to play a role in hyperalgesia and allodynia in animal studies [5].

#### Dysautonomia

Interestingly, the onset of symptoms was accompanied by diarrhoea lasting around 3 weeks in the new patient described in Section A. This is similar to a previously described ITPR1-IgG/anti-Sj-positive patient, in whom the onset of polyradiculoneuropathy was preceded by bowel and bladder dysfunction, which lasted several months [16]. That patient also exhibited pathological reduced heart frequency variability, a typical sign of autonomic neuropathy of the heart. Given that ITPR1 is expressed in the sympathetic ganglia as well as in the smooth muscle cells of the bowel and the bladder and that ITPR1-IgG/anti-Sj was shown to bind to smooth muscle cells in the enteric wall [1], a possible autoimmune pathogenesis cannot be excluded also in the present case. Diarrhoea is also a typical prodromal symptom in DPPX (dipeptidyl-aminopeptidase-like protein 6) syndrome, another novel antibody-related autoimmune disease of the CNS [34–36, 38], and paraneoplastic enteropathy is a well-recognized complication of cancer. As mentioned above, subacute dysautonomia (confirmed by thermoregulatory sweat test and autonomic reflex test) was also present in the sole previously reported patient with cognitive decline and serum ITPR1-IgG/anti-Sj antibodies [18]. Overall, dysautonomia has been reported in 4/19 (21%) ITPR1-IgG/anti-Sj-seropositive patients [16, 18, 27]. Studies evaluating the frequency of ITPR1-IgG/anti-Sj in patients with paraneoplastic or non-paraneoplastic autonomic enteropathy and other types of dysautonomia may thus be warranted.

Notably, repeated stroke episodes attributed to intermittent atrial fibrillation occurred in one patient with ITPR1-IgG/anti-Sj-associated dysautonomia [16]. ITPR1 is also present in human and murine cardiac myocytes [39–41] and has been implicated in modulating the propensity of the human myocardium to develop arrhythmias [39].

#### Further symptoms

In addition to the predominant cognitive deficits and neuropsychiatric symptoms, the patient described in Section A also developed transient symptoms of limb and gait ataxia, blurred vision for a period of around 6–8 weeks, and persisting tetraparesis. Interestingly, “weakness in extremities”, “subacute spastic paraparesis”, “extensive myelitis” and “blurred vision”, respectively, have been described in four further recently published ITPR1-IgG/anti-Sj-positive patients [18], again suggesting that neuronal damage or dysfunction in ITPR1-IgG/anti-Sj-associated disease may be more widespread than previously known.

#### CSF findings

Lumbar puncture was normal twice in our patient described in Section A, but on another occasion revealed blood–CSF barrier dysfunction (as indicated by an increased albumin CSF/serum quotient [QAlb]), which was also present in the only other patient with available data [16]. Including the present patient, CSF total protein, which strongly correlates with QAlb, was elevated in 4/7 seropositive patients. OCB were negative in 5/6 patients, including in 4 matched CSF/serum samples taken over a period of 4 months in one of them, and pleocytosis was present in 1/5. In one case, breast cancer cells were present in the CSF [18]; this highlights the need for detailed pathological examination of CSF cells in patients with PND.

#### Serological findings

All ITPR1-IgG/anti-Sj-seropositive patients analysed have been positive in recombinant CBA and have, in addition, shown the distinct binding to PC somata and dendrites described in the index patient when tested by immunohistochemistry using cerebellum tissue sections [1, 16, 18, 26, 27]. Reported CBA titres ranged between 1:320 and 1:245,760 (median 1:30,720; *N* = 13) in the serum and between 1:4 and 1:8192 (median 1:128; *N* = 5) in the CSF. IHC titres varied between 1:100 and 1:32,000 (median 1:3200; *N* = 7) in the serum; IHC CSF titres were reported only for two patients (1:250 and 1:1000, respectively). The CSF/serum ratio was reported for 6 patients (4 × CBA, 2 × IHC) and ranged between 0.004 and 0.033 (median 0.017; *N* = 6). While this could indicate intrathecal synthesis based on a normal CSF/serum ratio for total IgG of around 1/440, data on ITPR1-IgG-specific antibody indices (AI), which control for possible blood–CSF barrier dysfunction, were assessed only in two cases. In both cases the ITPR1-AI was below 4 (1 × based on IHC titres and on CBA titres, 1 × based on CBA titres), indicating an extrathecal origin of ITPR1-IgG [16]. In line with this finding, CSF-restricted OCB were absent in these two patients.

#### Radiological findings

Magnetic resonance imaging (MRI) showed cerebellar or pontocerebellar atrophy in 3 patients with ACA and available data; 1 additional patient showed several demyelinating supratentorial brain lesions (this patient had previously been considered to suffer from multiple sclerosis but steroids, interferon-beta and glatiramer acetate were all not effective); and 1 patient only ischaemic brain lesions. MRI was normal in 1. In three cases, MRI findings were not reported. Spinal cord lesions (longitudinally extensive in one) were noted in two patients, both of whom were diagnosed with myelopathy; in one of these, PNP was present in addition and MRI showed diffuse enhancement of the cervical nerve roots. Among patients with encephalitis/encephalopathy, a FDG-PET/CT revealed glucose hypermetabolism in the right medial temporal lobe (amygdala, parahippocampus) and in the basal ganglia was noted in 1; generalized brain atrophy on MRI (prominent in temporal lobes) in another; and subcortical right occipito-parietal T2-hyperintense lesions with gadolinium enhancement in a third patient.

#### EEG findings

EEG findings were not systematically reported. One patient with high-titre ITPR1-IgG serum antibodies developed a status epilepticus [18]; in a further high-titre patient (positive in both serum and CSF), “EEG-electrographic seizures” were documented [18]. In the patient reported in Section A, right parietal delta or theta activity and intermittent right temporal slowing was noted on several occasions, with intermittent normalization following immunotherapy. Of note, epileptic seizures have also been observed in mice lacking ITPR1 [42].

#### Tumour associations

In a substantial proportion of patients ITPR1-IgG/anti-Sj was found to be associated with tumours [1, 16, 18, 26, 28], some of which were demonstrated to express ITPR1 [16, 26]. ITPR1-IgG/anti-Sj-associated disease may thus be considered a facultative paraneoplastic neurological syndrome (PNS). Overall, a tumour was found in 9/20 (45%) of the ITPR1/anti-Sj-seropositive patients with available data (3 × breast, 1 × breast and endometrial, 3 × lung [including non-small lung cancer/adenocarcinoma in 2, not specified in 1], 1 × renal, 1 × multiple myeloma) [1, 16, 18, 26, 27]. However, this may underestimate the rate of paraneoplastic cases, given that (a) the tumour status was unknown in some patients, that (b) neurological manifestations may precede tumour diagnosis by many years in patients with paraneoplastic neurological disorder (PND) (as was indeed the case in one of the ITPR1-IgG/anti-Sj-seropositive index patients), and (c) that the underlying tumour not uncommonly remains occult in PND, often being found only upon autopsy. A positive CA125 test was noted in one additional case (see Section A), cervical dysplasia in another [18], and severe weight loss in a further one [18]. One additional case occurred 5 years after bone marrow transplantation for Fanconi anaemia, a genetic condition that confers a high risk of cancer [18]. According to the updated criteria for PNS [43], ITPR1-IgG/anti-Sj could be classified as “intermediate-risk antibody (associated with cancer in 30–70%)”.

In all but one case, the tumour was not known before onset of the neurological symptoms. One patient had a history of two cancers (breast and endometrial), but no information of tumour dissemination at the time of presentation was given [18]. Of 7 patients with a solid tumour and available data on tumour dissemination, five (71%) had metastases at the time of neurological presentation (3 × lymph nodes, 1 × bones and liver, 1 × brain). In one case, ITPR1-expressing breast cancer (associated with a germline mutation in the BCRA1 gene) was not found until 11 years after neurological onset, when it had already spread to the lymph nodes [26, 27].

Mastectomy and adjuvant tamoxifen led to tumour remission in the latter case, but the patient’s neurological status remained unchanged, probably reflecting early irreversible loss of PC, as also observed in other types of PND. In a patient with adenocarcinoma of the lung, removal of the tumour led to mild clinical improvement, but the patient was still unable to walk or stand [16]. No information on the effect of tumour removal is available for the remaining patients; however, two had died at last follow-up (1 × renal cancer with lymphadenopathy; 1 × metastatic breast cancer) [18].

#### Response to immunotherapy

Including the present case, the response to immunotherapy was reported for 10 patients (7 × PLEX, 7 × IVIG, 7 × IVMP/”IV steroids”/“steroids”, 1 × rituximab, 1 × glatiramer acetate and interferon beta-1a for suspected multiple sclerosis; 1 × cyclophosphamide). However, no significant effect was noted in most cases, with the exception of the patient reported in detail in the Appendix; the only other patient with cognitive decline, as mentioned above, who initially showed a good response to steroid treatment (but later progressed); and one patient with myelopathy in whom at least a short-lasting mild benefit from treatment with PLEX, IVIG and rituximab was found. As an important limitation, only very limited data on exact treatment regimens and, importantly, treatment duration were reported for most cases. Moreover, it cannot be excluded that the number of PLEX cycles was not sufficient to eliminate the antibodies in some cases; in one patient ITPR1-IgG/anti-Sj was still detectable at a titre of 1:1000 (CBA) 1 week after 7 cycles of PLEX [16]; no information on ITPR1-IgG/anti-Sj titres after PLEX treatment was reported for the remaining cases.

Four patients had died at the time of publication, but no information on the cause of death was reported, and no data was provided on the length of follow-up for most patients. One patient died 1 year after onset; at the time of neurological manifestation, he had already metastasis to the bones and liver from renal cell cancer; in another deceased patient, malignant breast cells were present in the CSF at the time of neurological onset. Eight patients were alive 2 years (mild dementia at last follow-up), 5 years (wheel-chair bound), at least 4 years (progressive with cognitive decline and seizures), at least 1.5 years (severe neurological sequelae), 23 months (PNP), 5 months (mild PNP, severe depression), 11.5 years (ACA, severely disabled), 5 months (ACA, no improvement), and 4 years (progressive ACA), respectively, after neurological onset.

#### Pathophysiology

It is still not known with certainty whether the antibody itself causes the symptoms present in patients with ITPR1 autoimmunity, since no passive transfer experiments, which would be required to demonstrate a direct pathogenic impact of ITPR1-IgG/anti-Sj, have been conducted so far. However, several lines of indirect evidence support the notion of an autoimmune pathogenesis at least in the case presented in Section A:

1. ITPR1, the target antigen of ITPR1-IgG, is expressed not only in PC but also in neurons in the cerebral cortex, the hippocampal formation and amygdala, and the temporal cortex, which renders encephalitis/limbic encephalitis a plausible complication of anti-ITPR1 autoimmunity, as well as in the basal ganglia [16, 17] (Fig. 1).
2. An FDG-PET scan performed during an episode of acute deterioration indeed demonstrated glucose hypermetabolism in the right medial temporal lobe (amygdala, parahippocampus), compatible with limbic encephalitis, and in the basal ganglia, and EEG repeatedly demonstrated theta and delta wave activity, including intermittent right-temporal slowing, during a relapse-like episode. Neuropsychologically, a diagnosis of a cognitive disorder consistent with right-temporal lobe damage was made.
3. Treatment with high-dose IVMP or a combination of PLEX, IVMP and IVIG, repeatedly resulted in significant and prompt clinical improvement, sometimes with complete recovery, as demonstrated by neuropsychological testing and confirmed both by the patient herself and her caregiver. On two occasions, immunotherapy was followed also by EEG normalization.
4. Similarly, immunosuppressive treatment with cyclophosphamide was paralleled by clinical improvement and relative stabilization of the disease course. Of note, the patient and her caregiver both reported that symptoms repeatedly worsened at the end of each cyclophosphamide cycle but soon improved after the next cyclophosphamide infusion, finally resulting in shortening of the treatment intervals.
5. ITPR1-IgG/anti-Sj remained clearly positive over the entire course of disease, with serum titres ranging between 1:100 and 1:1000 (cut-off ≥ 1:10).
6. ITPR1-IgG/anti-Sj was detectable also in the CSF during acute deterioration, although at low titres suggestive of passive diffusion from the peripheral blood into the CNS rather than intrathecal synthesis. In fact, clinically apparent infections (including respiratory infection, urinary tract infection and cholecystitis) or laboratory signs of inflammation, such as an increase in CRP levels or BSR, preceded or accompanied most episodes of clinical deterioration. Eleven of 13 CRP tests performed during the 1st year of disease yielded positive results (maximum 22.3 mg/l). This is of note, since peripheral infections may alter the blood–brain barrier (e.g., due to an increase in serum levels of proinflammatory cytokines, metalloproteinases, ROIs, etc.) and facilitate passive diffusion of serum IgG into the CNS. As mentioned above, acute deterioration was in fact associated with blood–CSF barrier disruption on one occasion (indicated by an elevated albumin CSF/serum ratio). Similarly, QAlb was also elevated twice in one of the only two other ITPR1-IgG-seropositive patients with published data [16]. Preceding infections are thought to trigger clinical attacks also in other autoantibody-associated disorders [44–46].
  In this context, it is important to underline that the absence of total IgG OCB and of intrathecal ITPR1-IgG/anti-Sj synthesis, as suggested by a negative AI, does not per se argue against a pathogenetic role of the antibody. Rather, negative OCB and negative AIs are frequent findings in antibody-related autoimmune disorders of the CNS, including MOG-IgG-positive encephalomyelitis [45–49] and AQP4-IgG-positive neuromyelitis optica spectrum disorders [44, 50], two diseases in which a direct pathogenic impact of the antibody has been proven or is highly likely [51–54]. A negative ITPR1-IgG-AI, indicating an extrathecal origin of CSF ITPR1-IgG/anti-Sj, was reported also in the only other patient investigated so far [16].
7. No co-existing anti-neural autoantibodies were present in addition to ITPR1-IgG/anti-Sj in the CSF and serum in our patient, despite very broad screening for such reactivities. Similarly, ITRP1-IgG/anti-Sj was absent in 80 neurological disease controls with autoantibody-related neurological syndromes or multiple sclerosis in three previous studies [1, 18, 27].
8. ITPR1-IgG/anti-Sj has been shown to be absent in healthy individuals (*N* = 250) [16, 18, 27] as well as in patients with non-neurological disorders (*N* = 105) [18, 27] upon testing by cell-based assays and was absent in almost 52,000 samples from patients with neurological symptoms of putative autoimmune aetiology in the 2018 study by Alfugham et al. [18]. This renders accidental coincidence at least unlikely.
9. Despite wide-ranging differential diagnostic endeavours, no other disorder that would better explain the patient’s symptoms was found.

It is of note that ITPR1-IgG/anti-Sj belonged exclusively to the IgG2 subclass in the present patient, in contrast to two of the index cases with ACA, which showed mainly IgG1 antibodies with only weak IgG2 and IgG3 reactivity (no data available from the third index patient) [1]. However, ITPR1-IgG/anti-Sj of exclusively the IgG2 subclass was also observed in two of the three patients with peripheral neuropathy previously reported by us [16].

Although complement activation by IgG1 and IgG3 is much stronger than that by IgG2, at least in humans (partly due to reduced binding of C1q to IgG2), IgG2 is still capable of triggering the classical complement pathway [55–58], putatively depending on antigen surface density [55–57], antibody concentration and hexamer formation [59]. IgG2 is also said to have limited Fc-receptor-mediated immune effector functions, because it binds only poorly to FcγRIIIa [60, 61]. However, myeloid cell-mediated antibody-dependent cellular cytotoxicity (ADCC) may be less affected than NK cell-mediated ADCC [57, 62].

Interestingly, all exclusively ITPR1-IgG2-positive patients (*N* = 3), the present case among them, were relatively mildly affected, while the exclusively [16] or mainly [1] ITPR1-IgG1-positive patients with available outcome data (*N* = 2) were severely disabled at most recent follow-up. IgG subclasses were not assessed in the remaining ITPR1-IgG/anti-Sj-seropositive patients reported so far [18, 26, 27]. IgG subclass analysis should be included in all future studies on ITPR1-IgG/anti-Sj, since differences in subclass distribution may be associated with pathogenetic and, in consequence, clinical and prognostic differences. Future studies should address the therapeutic and prognostic impact of differences in IgG subclass distribution.

ITPR1 is traditionally thought to be expressed mostly in the membrane of the smooth endoplasmic reticulum (ER) (and to a lesser degree on rough ER and the nuclear envelope). Therefore, it may not be accessible to serum or CSF ITPR1-IgG. If that is so, ITPR1-IgG/anti-Sj may just be a diagnostic marker, as is the case in other paraneoplastic neurological disorders, and T cell-mediated pathomechanisms may play a more important role. In a previous study, we demonstrated the presence of ITPR1-specific PBMCs in an ITPR1-IgG/anti-Sj-seropositive patient by use of a 3H-thymidine proliferation assay, including, besides ITPR1-specific B cells, CD4+ T cells and CD8+ CD45RO+ T cells [16].

On the other hand, surface localization of ITPR1 has indeed been reported to occur in some cell types, including neurons [19–25]. Moreover, uptake of human [63–72] as well as mouse [73] IgG autoantibodies targeting intracellular antigens by neurons has been described in numerous studies, as we have outlined elsewhere [2]. For example, incorporation of paraneoplastic anti-CDR2 and CDR2L antibodies (anti-Yo) has been reported to affect PC calcium homoeostasis [72] and to cause defective PC arborization [72] and PC cell death in cultured PCs [64]. Similarly, amphiphysin autoantibodies from patient sera transferred to rats were found to be internalized by neurons, to alter the function of inhibitory synapses in vivo [74], and to cause dose-dependent stiffness, with spasms resembling human stiff-person syndrome [74–76], as well as anxiety behaviour [77]. Autoantibodies to recoverin have been shown to induce caspase-dependent apoptosis after being taken up by retinal cells, probably via endocytosis [78]. A pathogenic effect has also been proposed for antibodies to the intracellular antigen GAD65 (glutamate decarboxylase 65) [79, 80]. Finally, endocytic uptake of autoantibodies has also been found for a subset of anti-DNA antibodies [81–84]. Of note, incorporation of IgG and IgM by PCs has been reported to occur independent of binding of the antibodies to PC surface antigens [65]. Taken together, these findings would imply that, at least with regard to some subsets of neurons, antigen surface expression may not be a prerequisite of antibody uptake and autoantibody-mediated functional or cellular cell damage [2]. ITPR1-IgG-related damage might then be mediated by disruption of Ca2+ homoeostasis via blocking or stimulation of ITPR1. This could result either in neuronal dysfunction (which would be compatible with the lack of radiological evidence for inflammatory brain damage or atrophy in the patient described in Section A and the partial—and once almost complete—recovery observed after immunotherapy in that patient) or, given that release of Ca^2+^ from the ER upon ITPR1 stimulation plays an important role in the induction of apoptosis [85–87], neuronal cell death. Further studies, including passive transfer experiments, are needed to shed more light on the pathogenetic relevance of ITPR1-IgG/anti-Sj.

Serum ITPR1-IgG/anti-Sj titres in the present patient were clearly positive (1:1000, 1:320, 1:100, respectively) but lower than those reported in two of the three index patients with cerebellar ataxia [1] and two of the three patients with neuropathy described in Ref. [2], all of whom were tested using the same methods as applied in the present case. However, no correlation of ITPR1-IgG/anti-Sj titres with the severity or type of clinical or oncological phenotype was found in a previous study [18], although more data from larger cohorts are certainly needed to draw definite conclusions.

## Discussion

ITPR1-IgG/anti-Sj autoantibodies in patients with neurological symptoms have been first described only relatively recently [1]. This new reactivity was initially thought to be associated mainly with autoimmune cerebellar ataxia. However, as shown in this review, the spectrum of syndromes associated with ITPR1-IgG/anti-Sj is much broader. A substantial proportion of all published cases (~ 45%) occurred in tumour patients, which renders the condition a facultative paraneoplastic disorder and the antibodies potential tumour markers.

In addition to providing a general overview of the clinical and paraclinical features and summarizing the current knowledge on the pathophysiology of this new disorder, we here specifically point to the association of serum ITPR1-IgG/anti-Sj with dementia and psychosis by reporting on a patient in whom these antibodies were associated with rapidly progressive, severe cognitive decline, mainly affecting memory, attention and executive functions, optic hallucinations and depression.

While this is the only detailed report on that association, neuropsychiatric symptoms have been described in a few further patient positive for serum ITPR1-IgG/anti-Sj, including dementia and encephalopathy [18], confusion [16], memory loss [16], insomnia, anxiety and depression [18], as well as temporal lobe atrophy [18] and suspected temporal lobe epilepsy [16]. This provides a rationale for investigating ITPR1 autoimmunity in patients with psychiatric symptoms or dementia of unknown cause as well as in patients with suspected limbic encephalitis.

Little is known so far in terms of optimum treatment of patients with ITPR1-IgG/anti-Sj-associated autoimmunity. It is therefore an important observation that immunotherapy repeatedly resulted in significant clinical improvement and in relative stability in the long-term in the patient described here. On the other hand, many ITPR1-IgG/anti-Sj-positive patients showed no or only little response to immunotherapy, and the reasons for differences in treatment response are not well understood. It is therefore of interest that a milder course of disease was associated with the exclusive presence of ITPR1-IgG/anti-Sj antibodies of the IgG2 subclass in some patients, which warrants systematic evaluation of IgG subclasses in ITPR1 autoimmunity. Moreover, ITPR1-IgG/anti-Sj was still detectable shortly after 7 cycles of PLEX in the only patient with available data. This might indicate a need for prolonged PLEX treatment (as already standard of care in many non-neurological autoimmune diseases) in some patients. When planning PLEX, it is import to consider that ITPR1-IgG/anti-Sj titres are very high in some cases (up to 1:245,760).

Future studies should also pay attention to less well-documented symptoms such as dysautonomia, bladder and bowel disturbances and pain as well as to spinal cord and optic nerve involvement.

## Conclusions

Our findings support the notion that the spectrum of symptoms associated with autoimmunity to ITPR1 in neurological patients is broader than initially thought. They provide a rationale for studies evaluating the frequency of ITPR1-IgG/anti-Sj in patients with cognitive decline and/or psychosis of unknown aetiology. In many cases, the presence of ITPR1-IgG was first suggested by IHC. This highlights the importance of screening sera and CSF from patients with suspected autoimmune encephalitis by means of tissue-based assays in order to recognize reactivities that may prompt a second level screening for ITPR1 or other rarer antigens by means of antigen-specific assays, considering that many laboratories routinely use only commercial assays (blot or CBA) for the most common anti-neuronal and anti-glial antibodies. Passive transfer experiments are needed to clarify whether the disease is directly caused by the antibody or rather by T-cell-mediated mechanisms. Future studies investigating treatment responses and outcome in ITPR1-related autoimmunity should systematically evaluate ITPR1-IgG/anti-Sj subclasses, given that the presence of exclusively IgG2 antibodies was associated with a milder course of disease in some cases compared to patients with predominantly IgG1 antibodies. Considering the high rate of paraneoplastic cases, tumour screening seems essential in patients presenting with ITPR1-IgG/anti-Sj-related autoimmune encephalitis or polyneuropathy.

## Figures and Tables

**Fig. 2:**
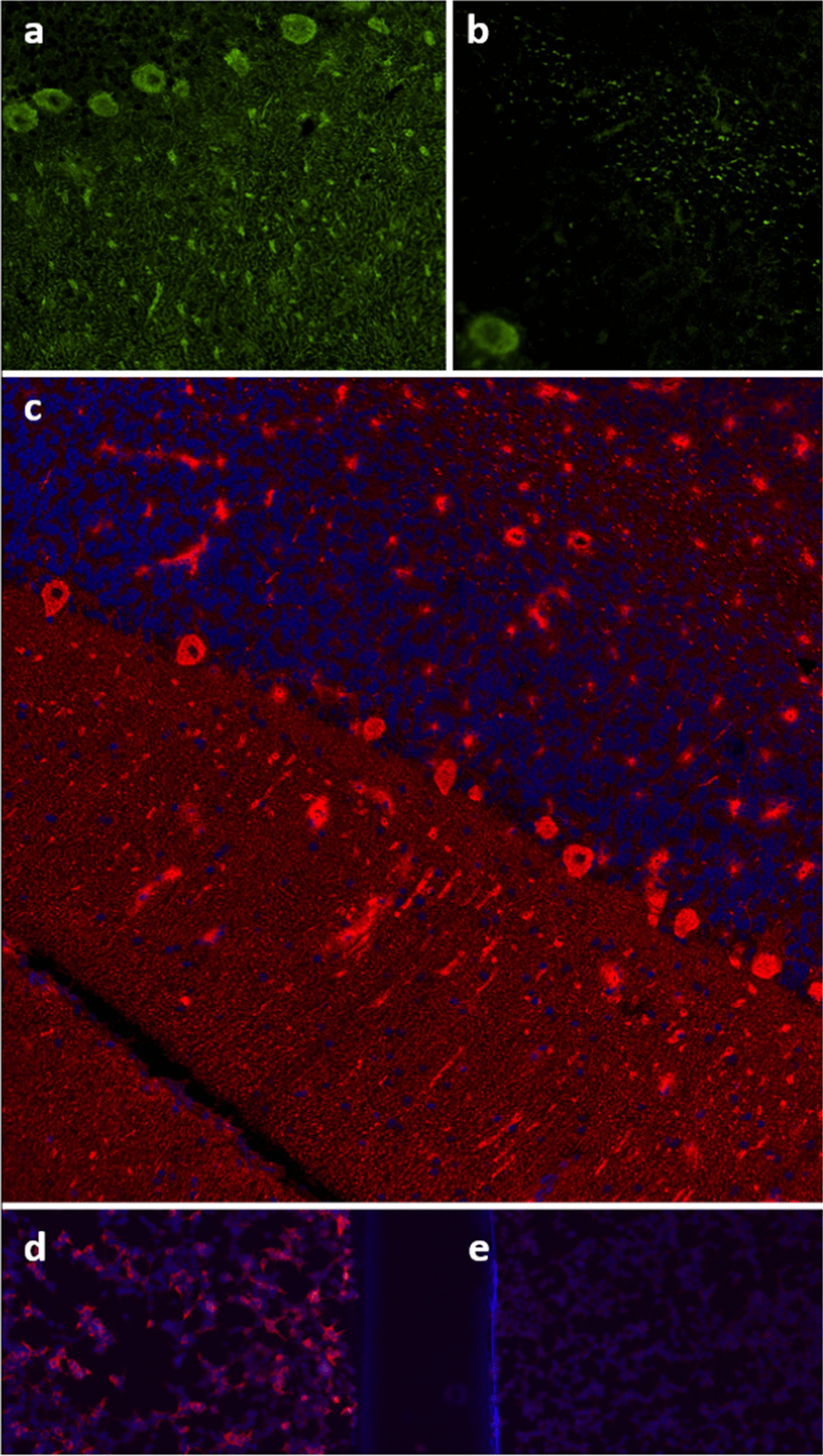
Binding of ITPR1-IgG/anti-Sj to cerebellar sections (**a**–**c**; **a**: staining of the Purkinje cell layer and the molecular layer; **b**: staining of axonal cross sections in the cerebellar white matter) and to ITPR1-transfected HEK293 cells (**d**) but not to mock-transfected HEK293 cells (**e**). Green fluorescence (FITC) indicates binding of patient IgG; red fluorescence (AF568) indicates binding of patient IgG of the IgG2 subclass; blue fluorescence corresponds to cell nuclei stained by 4′,6-diamidino-2-phenylindole. Note that the vessel-associated staining present in panel (**c**) is caused by non-specific cross-reactivity of the detection antibody with primate IgG that has penetrated the vessel walls post mortem during preparation of the brain specimen, was therefore present also after incubation of the sections with PBS instead of serum (not shown), and does not indicate the presence of anti-vessel antibodies in our patient; no such cross-reactivity was seen when using another, whole-IgG-specific secondary antibody (**a**, **b**)

## Data Availability

All data generated or analysed during this study are included in this published article.

## Appendix

In this appendix, we report an ITPR1-IgG/anti-Sj-positive patient with progressive cognitive decline, affecting mainly short-term memory, executive dysfunction, attention deficits, a sleep disorder, and psychotic symptoms. Most previous cases have been described only very briefly. Given the rarity and the relative novelty of the disease, we decided to give a comprehensive description of the patient’s clinical and paraclinical findings to draw a more vivid ‘real-life’ picture of the disorder than statistical analyses alone can provide. Moreover, only detailed case descriptions allow evaluation of treatment responses and outcomes in a meaningful way.

### Initial presentation and serological findings

A 48-year-old saleswoman with no pre-existing conditions except for euthyroid struma (treated by thyroidectomy more than 30 years earlier), cholecystolithiasis, type 2 diabetes and hypertension and no history of drug abuse first noticed increasing forgetfulness in summer 2019. At the end of September 2019, her family noted rapidly progressive short-term memory deficits interfering with everyday activities (e.g., missed appointments, forgetting to switch off the oven and iron), confused actions (cleaning of the floor with oil instead of water; inability to switch on the computer; dressing wet after showering) with a tendency to trivialize the errors made, and other personality changes (more quiescent and secluded, uncommunicative and taciturn, increased need for sleep; emotional blunting, less empathetic, increased irritability; at times sudden and impulsive agitation, newly occurring buying frenzy), which led to referral to a neurological hospital a month later.

On admission at the end of October 2019 the patient was fully conscious, oriented to person, time, place, and situation, and cooperative, and no focal neurological deficits were present except for bilateral dysdiadochokinesis. However, flat affect, increased irritability and perseverations were noted, and neuropsychological testing revealed severe cognitive impairments affecting concentration/attentional control, attention span, shared attention, short-term memory, working memory, long-term memory, processing speed, action planning and problem/error recognition (with constant perseveration of previous errors), leading to a diagnosis of ‘dysexecutive dementia’ (Mini-Mental Status Examination [MMSE] 27/30 [0/3 terms recalled], clock-drawing test [CDT] 3/7, DemTect [88] 9/18). The results of contrast-enhanced cranial MRI carried out a few days prior to admission were normal. Lumbar puncture performed on the day of admission showed a normal cell count, negative oligoclonal bands (OCB) [89], no qualitative evidence of intrathecal IgG, IgA or IgM synthesis, a normal albumin CSF/serum ratio and normal CSF total protein, CSF L-lactate and CSF glucose levels. However, serological screening for paraneoplastic and non-paraneoplastic antibodies demonstrated strong anti-neuronal serum reactivity characterized by distinct staining of the dense network of PC neurites in the molecular layer of the cerebellum, the PC somata, the main PC dendrite branches and the PC axons passing through the granular layer and cerebellar white matter (Fig. 2). An identical binding pattern was found with primate (1:320), rat (1:320) and mouse cerebellum (1:100) sections. The presence of serum autoantibodies to ITRP1 was demonstrated by use of a cell-based assay (CBA) employing ITPR1-transfected and mock-transfected HEK293 cells as test substrates [1, 16] (cells obtained from Euroimmun, Lübeck, Germany) (1:1000) (Fig. 2). The antibodies belonged exclusively to the IgG2 subclass; neither antibodies of the IgG1, IgG3 or IgG4 subclasses nor IgM or IgA antibodies to ITPR1 were detected by the immunohistochemical assay or by the CBA. A matched CSF sample was positive for ITPR1-IgG/anti-Sj in the CBA when tested undiluted, but was negative when tested at dilutions that would suggest intrathecal synthesis (as indicated by an antibody index [AI] ≥ 4), taking into account Qlim [90]. The findings of routine laboratory tests were normal except for mildly elevated serum C-reactive protein (CRP) (7 mg/l; normal: < 5) and blood sedimentation rate (22 mm/h). Electroencephalography (EEG) showed intermittent right-sided frontoparietal delta wave activity. Broad screening for other anti-neural autoantibodies (anti-Hu, -Yo, -Ri, -ANNA-3, -Tr/DNER, -Ma2/Ta, -amphiphysin, -GAD65, -NMDAR, -CASPR2, -LGI1, -AMPAR, -CV2/CRMP5, -SOX1, -mGluR1, -mGluR5, -GABA-A-R, GABA-B-R [91, 92], -Homer-3, -CARPVIII, -GluR-delta2, -ARHGAP26, -DPPX, -Zic4, -recoverin, -titin, -neurochondrin, -flotillin [93], -IgLON5, -glycine receptor, -AQP4, -MOG, -myelin), for systemic or organ-specific rheumatological autoantibodies and for anti-thyroidal autoantibodies (anti-TPO, anti-thyroglobulin) was negative, as was testing for neurolues, neuro-HIV, Creutzfeldt–Jakob disease, neurosarcoidosis and neuroborreliosis. Immunohistochemistry did not reveal evidence for additional anti-neural antibodies, irrespective whether primate, murine or rat CNS tissue was used. Testing of neuronal cell cultures, which is available at some research institutions, was not performed. Later the same day she left the ward in a disoriented state and was subsequently referred to a psychiatric hospital for risk of self-harm and impaired impulse control. There, besides marked impairment of short- and intermediate-term (but not biographic) memory and attention, also a significant loss in the capacity to judge and discern as well as behavioural abnormalities (e.g., ‘reading’ books holding them upside down) were documented (MMSE 23/30). CRP remained elevated (7.7 mg/l; 9.3 mg/l). A second (non-contrast-enhanced) MRI performed 3 weeks after the patient’s first admission was normal as well, as was a second lumbar puncture (0 cells/µl, no OCB, normal albumin CSF/serum ratio, negative HSV PCR and VZV PCR, negative measles virus AI). EEG showed right-sided frontoparietal delta waves. Considering the serological findings, a diagnosis of possible autoimmune encephalitis was made and treatment with high-dose IVMP (5 × 1 g/day) was commenced. This was followed by marked clinical improvement with almost complete resolution of the patient’s cognitive and psychiatric symptoms (MMSE 29/30). After discharge at the end of November 2019, she went back to work in her previous job, complaining only of mild residual cognitive and physical fatigue. According to her husband, she was “her old self again”.

Repeat neuropsychological testing in mid-January 2020 confirmed marked and sustained improvement (MMSE 29/30 [3/3 terms recalled], CDT 7/7, DemTect 16/18; normalized affect and executive functions with restored problem recognition and solution capacity; only mild residual attention deficit). Cranial MRI and CSF analysis (no pleocytosis, OCB, intrathecal immunoglobulin synthesis, or blood–brain barrier dysfunction) were again normal. Routine laboratory tests were unremarkable except for an increase in CRP levels (13 mg/l; normal: < 5). EEG showed right-sided parieto-occipital theta wave activity. Serological testing again revealed high-titre ITRP1-IgG serum autoantibodies (CBA 1:1000; IHC 1:1000 on rat tissue, 1:320 on primate tissue, 1:100 on mouse tissue); as at first testing, only IgG2 antibodies were present. Incubation with undiluted CSF now resulted in slightly more distinct binding of IgG (in particular IgG2) to the ITPR1-transfected cells, but again no evidence for intrathecal ITPR1-IgG/anti-Sj synthesis was found. Again, a broad spectrum of other anti-neural antibodies was tested in both serum and CSF (anti-Hu, -Yo, -Ri, -ANNA-3, -Tr/DNER, -Ma2/Ta, -amphiphysin, -GAD65, -NMDAR, -CASPR2, -LGI1, -AMPAR, -CV2/CRMP5, -SOX1, -mGluR1, -mGluR5, -GABA-A-R, -GABA-B-R, -Homer-3, -CARPVIII, -GluR-delta2, -ARHGAP26, -DPPX, -Zic4, -recoverin, -titin, -neurochondrin, -flotillin, -IgLON5, -glycine receptor, -AQP4, -MOG, -myelin) but all results were negative. High-dose IVMP therapy (3 × 1 g/day) was repeated, followed by oral tapering, with the intention of eliminating the mild residual deficits and achieving long-term stability.

### First relapse

Around 2 weeks later, however, while still being treated with oral prednisolone (60 mg/day), the patient contracted a respiratory infection, which was treated with amoxicillin. This was followed by a severe relapse a further 10 days later in February 2020, characterized by a marked decline in cognitive function (MMSE 24/30, CDT 1/7, DemTect 8/16), again particularly affecting executive functions, short-term memory (markedly impaired with false-positive contents/confabulation) and attention, increased distractibility, psychomotor restlessness, disorientation, wandering, confusion (attempts to empty a washing basket with nothing in it), impaired impulse control/disinhibition, hypergraphia, logorrhoea, perseverations and, for the first time, optic hallucinations (handing over of non-existent letters). A neuropsychological diagnosis of “right-temporally accentuated cognitive disorder” was made. CRP and blood sedimentation rate were not elevated at the time of readmission. Lumbar puncture was again normal (no pleocytosis, normal cytology, no OCB, no malignant cells), as was MRI of the brain and cervical spinal cord. Notably, however, cranial FDG-PET revealed elevated cerebral glucose metabolism involving the right medial temporal lobe (amygdala, parahippocampal gyrus) and the right striatum, especially the putamen. EEG again showed right-sided parieto-occipital theta wave activity. Broad screening for classical paraneoplastic and non-paraneoplastic anti-neural autoantibodies (anti-Hu, -Yo, -Ri, -Ma2/Ta, -NMDAR, -AMPAR, -LG1, -CASPR2, -GABABR, -DPPX, -GAD65/67, -amphiphysin, -SOX1, -GluR-delta2) and CTD-related antibodies was again unremarkable, but ITPR1-IgG/anti-Sj was still detectable in the serum at a CBA titre of 1:1000 and also in the CSF at a titre of 1:4. Assuming an ITPR1-IgG/anti-Sj-related pathogenesis, the patient was then treated with 5 cycles of PLEX (removed plasma from the first cycle tested positive for ITPR1-IgG/anti-Sj at a CBA titre of 1:320), followed by a third pulse of high-dose IVMP (5 × 1 g/day; with oral tapering until July). This resulted (after an initial IVMP-induced psychotic episode) in significant improvement of the patient’s cognitive status, especially of attention, memory and psychomotor activity. Due to the absence of complete recovery, treatment with intravenous immunoglobulins (IVIG) (4 × 35 g/day) and anti-psychotic therapy with risperidone, pipamperone and lorazepam was added, resulting in improved impulse control and a reduction in psychomotor restlessness. One month later, neuropsychological testing confirmed marked improvement, with mild residual impairment of attention and processing speed (MMSE 28/30, CDT 3/7, DemTect 14/18). EEG was now normal.

### New symptoms

While a high proportion (9/20; 45%) of the ITPR1-IgG/anti-Sj-seropositive patients reported so far had acute cerebellar ataxia, no signs compatible with cerebellar involvement were noted in the present case during the first 10 months after onset, except for dysdiadochokinesis, which was apparent already at first presentation. At the end of March 2020, however, bilaterally pathological knee–heel (still present in July 2020) and finger–nose tests were found, and the patient developed an unsteady gait and mild tetraparesis (upper extremities: 4/5, lower extremities: 4/5; apparent also on arm and leg extension tests), prompting her to use a rollator for long walks. Cranial MRI performed in October 2020 and July 2021 again showed no cerebellar atrophy or any signs of cerebellitis. Serum ITPR1-IgG/anti-Sj was positive at a CBA titre of 1:100. At the most recent follow-up in August 2021, the patient’s gait instability and limb ataxia had resolved but she still used a rollator due to paraparesis. In April to May 2021, the patient in addition experienced an episode of blurred vision in both eyes lasting for 6–8 weeks, followed by complete recovery; no VEP was performed.

### Second relapse

It was decided that regular PLEX treatments should be carried out. However, the next cycle of PLEX had to be interrupted after the first treatment due to a severe allergic reaction. Long-term therapy with cyclophosphamide was initiated instead, starting at the beginning of May 2020 (350 mg/m^2^/day for 3 days). This was paralleled by further improvement of the patient’s cognitive test results (MMSE 30/30, CDT 7/7, DemTect 15/18), although some impairment of attention, memory and processing speed persisted. Serum ITPR1-IgG/anti-Sj was detected at a CBA titre of 1:100 immediately after the first cyclophosphamide cycle.

In October 2020, around a month after the third cyclophosphamide cycle, another relapse-like episode occurred, with increasing attention and memory deficits, disorientation, psychomotor restlessness and formal thought disorders, associated with increased CRP (13 mg/l; normal < 5) but no leucocytosis or fever. EEG showed intermittent right-temporal slowing but no typical epileptiform discharges. Cranial MRI was unremarkable. Treatment with IVMP (5 × 1 g/day, with oral tapering), risperidone, and lorazepam was followed by significant neurocognitive improvement and complete psychiatric recovery at re-evaluation in November as well as EEG normalization.

Four further cycles of cyclophosphamide were given in November 2020 and in January, April and June 2021; these were paralleled by relative stability. The patient herself noted a mild increase in symptom severity shortly before each cyclophosphamide cycle. She also underlined at that time that she felt the previous IVMP therapies had been “highly effective”. Serum ITPR1-IgG/anti-Sj remained detectable at a CBA titre of 1:320 (November 2020).

### Third (mild) relapse

In mid-July 2021 the patient was re-admitted with a mild depressive disorder associated with a mild subjective decline in short-term memory and executive functions (with no change observed upon neuropsychological testing). Lumbar puncture showed mild blood–CSF barrier dysfunction and, accordingly, slightly elevated CSF IgG and IgM levels, but no qualitative or quantitative evidence of intrathecal immunoglobulin synthesis. Brain MRI with gadolinium enhancement was normal. Treatment with IVMP (3 × 1 g/day) and sertraline resulted in prompt resolution of the patient’s symptoms. ITPR1-IgG/anti-Sj was still detectable in August at a titre of 1:1000 (CBA). In September 2021, it was planned to end the treatment with cyclophosphamide to avoid potential side effects of long-term use and to commence treatment with azathioprine.

### Dementia/neurodegeneration markers

CSF beta-amyloid 1–40 levels were elevated on two occasions in November 2019 but normal in January 2020; CSF beta-amyloid 1–42 levels were normal when tested at first admission and in November 2019, as well as in January and February 2020; the CSF beta-amyloid 1–42/1–40 ratio was determined three times and was always normal. CSF tau protein levels were mildly elevated in February 2020 (368 ng/l) but normal in November 2019 and January 2020; phospho-tau levels were elevated in November 2019 (78 ng/l) but normal at first presentation and again in January and February 2020.

### Tumour screening

ITPR1-IgG/anti-Sj-related autoimmunity has been reported to occur in a paraneoplastic context in some patients, but no tumour has yet been identified in the present case (last follow-up: August 2021). No M gradient was present; serum immune fixation and CSF isoelectric fixation, total serum IgG, IgA and IgM values, serum free kappa (*k*) and lambda (*l*) light chains, and the *k*/*l* quotient were all normal. In January 2020, tumour markers were tested. While the serum levels of CEA, CA19-9, SCC and NSE were normal, CA125 serum levels were markedly elevated (111 U/ml, normal: < 35; 88 U/ml in February 2020; 35 U/ml in August 2021). CT performed in November 2019 had shown bilaterally small nodular abnormalities in the lung (< 6 mm) as well as cystic structures at the isthmus uteri, both considered to be of unknown significance. Whole-body FDG-PET–CT (February 2020) was unremarkable in that regard, as were thoracic and abdominal follow-up CT (April 2021), pelvic MRI (February 2020), gynaecological examinations (November 2019 and February 2020), sonography of the abdomen (October 2019, February and April 2020; suggestive of chronic cholecystitis), and several chest X-rays [94].

## Abbreviations

ACA: Autoimmune cerebellar ataxia
ADCC: Antibody-dependent cellular cytotoxicity
AI: Antibody index
ART: Autonomic response testing
BSR: Blood sedimentation rate
CA125: Cancer antigen 125
CBA: Cell-based assay
CDT: Clock-drawing test
cMRI: Cranial MRI
CSF: Cerebrospinal fluid
CRP: C-reactive protein
CT: Computed tomography
CTD: Connective tissue disorders
CYCL: Cyclophosphamide
ER: Endoplasmic reticulum
EEG: Electroencephalography
EMG: Electromyography
FDG-PET: 2-Deoxy-2-[18F]fluoro-d-glucose positron emission tomography
FITC: Fluorescein isothiocyanate
Gd: Gadolinium
GFAP: Glial fibrillary astrocytic protein
HEK293: Human embryonic kidney 293 cells
IgG/M/A: Immunoglobulin G/M/A
IHC: Immunohistochemistry
ITPR1: Inositol 1,4,5-trisphosphate receptor 1
IVIG: Intravenous immunoglobulins
IVMP: Intravenous methylprednisolone
LETM: Longitudinally extensive transverse myelitis
MMSE: Mini-Mental Status Examination
MRI: Magnetic resonance imaging
NMDAR: *N*-Methyl d-aspartate receptors (NMDAR)
OCB: Oligoclonal bands
PC: Purkinje cells
PET–CT: Combined positron emission tomography and computed tomography
PLEX: Plasma exchange
QAlb: Albumin CSF/serum ratio
REM: Rapid eye movements
TST: Thermoregulatory sweat test
